# Role of Apolipoprotein E in Anxiety

**DOI:** 10.1155/2007/91236

**Published:** 2007-07-11

**Authors:** Jacob Raber

**Affiliations:** Departments of Behavioral Neuroscience and Neurology, Division of Neuroscience, Oregon National Primate Research Center (ONPRC), Oregon Health & Science University, L470, 3181 SW Sam Jackson Park Road, Portland, OR 97239, USA

## Abstract

Anxiety is most common among Alzheimer's disease (AD) patients with an age at onset under age 65. Apolipoprotein E4 (apoE4) is a risk factor for developing AD at an earlier age and might contribute to this effect. In mice, apoE plays a role in the regulation of anxiety, which might involve histamine receptor-mediated signaling and steroidogenesis in the adrenal gland. In addition, human apoE isoforms have differential effects on anxiety in adult mice lacking apoE and probable AD patients. Compared to wild-type mice, mice lacking apoE and apoE4 mice showed pathological alterations in the central nucleus of the amygdala, which is involved in regulation of anxiety. ApoE4, but not mice lacking apoE, or apoE3 mice showed impaired dexamethasone suppression of plasma corticosterone. Understanding how apoE modulates measures of anxiety might help the developments of therapeutic targets to reduce or even prevent measures of anxiety in health and in dementing illnesses.

## 1. INTRODUCTION

Noncognitive behavioral changes are the major cause of institutionalization of AD patients and a major concern for their caregivers [1–3]. Such changes are also a negative predictor of survival and quality of life for AD patients and contribute to increased costs [4, 5]. However, they have received much less attention than cognitive impairments. Most pharmacological strategies for controlling behavioral changes, including treatment with benzodiazepines, cause deterioration in mental performance and motor function [6]. In AD, anxiety is inversely related to mini-mental state examination (MMSE) score (i.e., worse with more severe dementia) [7]. Anxiety symptoms occur in about 50% [8] to 75% [9] of AD patients. A study describing the relationship between anxiety and nighttime behavioral disturbance in a community dwelling sample of 153 AD patients revealed symptoms of anxiety and patient awakening associated with higher levels of patient anxiety and patient impairments in activities of daily living (ADL) in 56% of the patients [10]. Individual-patient anxiety symptoms were risk factors for patient awakenings [10]. Anxiety symptoms become more common as the disease progresses and are associated with greater disability in daily activities [7, 8]. In more than half of the cases, the caregivers demand therapeutic intervention regardless of the effects on cognitive and motor function. Therefore, there is a need to better understand the mechanisms underlying increased anxiety and to develop better treatments for these conditions.

## 2. APOE, BRAIN FUNCTION, AND AD

ApoE plays an important role in the metabolism and redistribution of lipoproteins and cholesterol [11]. The three major human apoE isoforms are encoded by distinct alleles (*ε*2, *ε*3, and *ε*4). Compared with *ε*2 and *ε*3, *ε*4 increases the risk of cognitive impairments and of developing AD [12]. This increased risk might involve a loss of trophic function of apoE4 or gain of toxic function of apoE4. Anxiety is most common among AD patients with a younger age at onset (under age 65) [7]. ApoE4 is a risk factor for developing AD at an earlier age [12] and might contribute to this effect.

## 3. ROLE OF APOE IN REGULATING MEASURES OF ANXIETY REVEALED IN *APOE* ^–/–^ MICE

**Figure 1:**
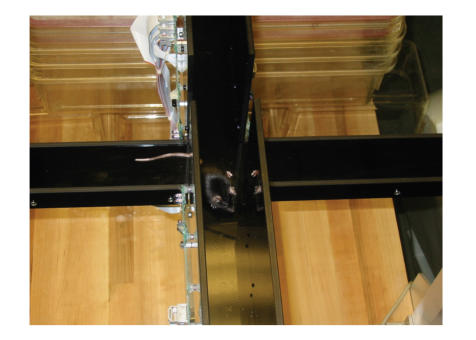
Elevated plus maze: the mice are tested for 10 minutes; while they are curious to explore the open areas, they are anxious to do so.

The elevated plus maze can be used to assess measures of anxiety in mice (Figure 1). The plus maze consists of a perpendicular cross elevated above the floor. The sides of one axis are walled off. There are infrared photobeams to record movements. Mice will prefer the safety of the enclosed, darker arms, but they like to explore the open arms and poke over the edge. Less anxious mice will venture more onto the open arms, and poke their heads more over the edges of the open arms. Male *Apoe* ^−/−^ (C57BL/6J-Apoe^tm1Unc^) and wild-type C57BL/6J mice were obtained from the Jackson Laboratory (Bar Harbor, Me). When measures of anxiety in the elevated plus maze were assessed in 6-month-old *Apoe* ^−/−^ male and wild-type control mice, *Apoe* ^−/−^ mice showed increased measures of anxiety [13]. These changes are age-dependent and not seen in 3-month-old mice.

## 4. APOE AND ADRENAL STEROIDOGENESIS

Liver and brain are the major sites of apoE synthesis. However, many other tissues, particularly steroidogenic tissues such as the adrenal gland, also express apoE [14, 15]. Adrenal apoE expression is the highest in cortical cells that synthesize glucocorticoids (GCs), declines when steroidogenesis is stimulated, and increases when it is blocked [14, 15]. The function of apoE synthesized by the adrenal gland is unclear. *Apoe* ^−/−^ mice show an age-dependent dysregulation of the hypothalamic-pituitary-adrenal (HPA) axis. The HPA axis regulates the secretion of GCs through a mechanism that primarily affects the adrenal gland. In *Apoe* ^−/−^ mice, activation of the HPA axis is seen at the same time as increased measures of anxiety are observed in the elevated plus maze. *Apoe* ^−/−^ mice have an age-dependent increase in basal adrenal corticosterone content and abnormally increased plasma corticosterone after restraint stress and anxiety testing in the elevated plus maze [13]. *Apoe* ^−/−^ mice also show increases in lipid droplets in adrenal cortex and medulla [13]. These data are consistent with hypersecretion of adrenal corticosterone and increased adrenal corticosterone content and with the reported inverse relationship between the levels of apoE mRNA and adrenal steroidogenesis, and they suggest a key role for apoE in the tonic inhibition of steroidogenesis and adrenal cortical activity.

## 5. HISTAMINE AND ANXIETY OF *APOE* ^–/–^ MICE

Histamine increases measures of anxiety [16], and altered histamine signaling could contribute to increased measures of anxiety in adult *Apoe* ^−/−^ male mice. We began to assess the possible role of histamine receptor-mediated signaling in regulating measures of anxiety in *Apoe* ^−/−^ and wild-type mice. Drugs were dissolved in saline and administered by intraperitoneal injection 1 hour before behavioral testing at the indicated doses, selected based on preliminary studies. The person testing the mice was blinded to genotype and treatment. The histamine H_3_ receptors modulate histamine release and synthesis via negative feedback. We assessed whether young (3–5-month old) *Apoe* ^−/−mice^, which show similar measures of anxiety in the anxiety-provoking open arms of the plus maze, respond differentially to histamine H_3_ receptor ligands. Anxiety levels were assessed 1 hour after intraperitoneal administration of thioperamide (5 mg/kg) or saline. Wild-type mice treated with H_3_ receptor antagonist thioperamide showed increased measures of anxiety as compared to wild-type mice treated with saline [17]. The total activity in the closed arms was comparable in the saline- and thioperamide-treated wild-type mice. These data indicate that the differences in measures in the open arms were not caused by differences in activity levels. In contrast, thioperamide had no effect on measures of anxiety in *Apoe* ^−/−^ mice.

Next we determined whether in wild-type and *Apoe* ^−/−^ mice, H_3_ antagonists also have differential effects on novel object recognition [18, 19]. During the training session, mice were allowed to explore for 15 minutes an open field containing two objects. For the retention session (24 hours later), they were placed back into the same open field for 15 minutes, after one of the familiar objects had been replaced with a novel object and the other familiar object with an exact replica. The percentage of time the mice spent exploring the novel versus the familiar object relative to the total amount of time they explored either object in the retention session was used as a measure of object recognition memory. Wild-type and *Apoe* ^−/−^ mice received saline, thioperamide (5 mg/kg), or clobenpropit (10 mg/kg) during the training and retention sessions [17]. The recently cloned H_4_ receptor [20] was found to have an affinity for H_3_-specific ligands. To rule out the possible contribution of the H_4_ receptor to the effects of thioperamide, we also treated wild-type and *Apoe* ^−/−^ mice with clobenpropit, a H_3_-specific antagonist that was reported to be an H_4_ receptor agonist as well [20]. In the training session, all groups of mice spent a comparable amount of time exploring each object. In the retention session, the saline-treated wild-type and *Apoe* ^−/−^ mice spent significantly more time exploring the novel object (wild-type: 8.37 ± 0.93 seconds; *Apoe* ^−/−^: 9.34 ± 2.76 seconds) than the familiar object (wild-type: 5.43 ± 0.69 seconds; *Apoe* ^−/−^: 5.15 ± 1.25 seconds), whereas the thioperamide- and clobenpropit-treated wild-type and *Apoe* ^−/−^ mice spent equal amounts of time exploring both objects. The similar effects of thioperamide and clobenpropit on novel object recognition indicate that they are mediated by the H_3_ receptor and not the H_4_ receptor.

To determine whether H_3_ ligands have differential effects on emotional learning and memory in wild-type and *Apoe* ^−/−^ mice, passive avoidance learning was used. Mice were placed in a lighted compartment of a chamber also containing a dark compartment. They entered the dark compartment by preference where they received a brief and slight foot shock (0.3 mA for 1 second). After 24 hours, the mice were again placed in the light compartment, and the time to reenter the dark compartment was measured. Drugs were administered 1 hour before behavioral testing on both days. Both saline- and thioperamide- (5 mg/kg) treated wild-type and *Apoe* ^−/−^ mice showed emotional learning and memory as the time to reenter the dark chamber was significantly higher on day 2 than day 1. There was no effect of thioperamide but consistent with increased measures of anxiety of *Apoe* ^−/−^ mice in the elevated plus maze, the latency to enter the dark compartment on day 1 was lower in *Apoe* ^−/−^ than wild-type mice (*P* < .05, Tukey-Kramer).

To determine whether there are differences in H_3_ receptor expression in young *Apoe* ^−/−^ and wild-type mice (3–5-month old) which could have contributed to their differential response to H_3_ antagonists on measures of anxiety, we performed saturation analysis with [^3^H]-N*α*-methylhistamine (NAMH) in brain regions that have been implicated in cognition or emotion [17]. The total number of receptors (B_max_ in nM) in the amygdala (wild-type: 87.3 ± 2.5; *Apoe* ^−/−^: 81.8 ± 2.3), cortex (wild-type: 119.9 ± 3.0; *Apoe* ^−/−^: 56.8 ± 5.8), and hippocampus (wild-type: 108.4 ± 10.5; *Apoe* ^−/−^: 29.1 ± 1.7) was lower in *Apoe* ^−/−^ than in wild-type mice. In the hypothalamus, B_max_ was not different between the groups. There was no difference in the binding affinities of [^3^H]-NAMH in any brain region. Thus, there is no simple association between levels of H_3_ receptor expression in structures associated with anxiety versus cognition, which could explain why H_3_ antagonists impaired hippocampus- and cortex-dependent novel object recognition [18] but did not increase more amygdala-dependent measures of anxiety in the plus maze.

In experimental models of anxiety, stimulation of H_1^−^_, but not of H_2^−^_, receptors increases measures of anxiety [16, 21, 22]. In *Apoe* ^−/−^ mice, reduced negative feedback via H_3_ receptors could increase histamine release and signaling of H_1_ and H_2_ receptors. To determine whether in *Apoe* ^−/−^ mice increased signaling of these receptors contributed to the increased measures of anxiety, 3–5-month-old wild-type and *Apoe* ^−/−^ mice were assessed in the elevated plus maze 1 hour after intraperitoneal administration of the H_1_ antagonist mepyramine (5.6 mg/kg), the H_2_ antagonist zolantidine (10 mg/kg), or saline. *Apoe* ^−/−^ mice treated with mepyramine, but not with zolantidine, showed reduced measures of anxiety as compared to *Apoe* ^−/−^ mice treated with saline [17]. The total activity in the closed arms was comparable and not significantly different between the saline-, mepyramine-, and zolantidine-treated *Apoe* ^−/−^ mice, indicating that the differences in measures in the open arms were not caused by differences in activity levels. In contrast, mepyramine had no effect on measures of anxiety in wild-type mice. The lack of an effect of H_1_ receptor blockade on measures of anxiety in wild-type C57Bl/6J mice is consistent with the lack of effect of H_1_ receptor blockade on measures of anxiety in wild-type ddY mice and it supports that the H_1_ receptor becomes activated at higher levels of histamine release [21]. The reduced measures of anxiety in *Apoe* ^−/−^ mice after H_1_ receptor blockade are consistent with the reported antagonizing effects of mepyramine on experimental anxiety induced by histamine releasers [16, 21] and the anxiogenic effects of the H_1_ receptor agonist and H_3_ receptor antagonist betahistine [22].

In *Apoe* ^−/−^ mice, the effects of mepyramine on measures of anxiety in the plus maze were not associated with a reduced HPA axis response. Plasma ACTH and corticosterone levels were assessed directly after plus-maze testing [23]. Compared to saline controls, mepyramine reduced the plasma corticosterone levels in wild-type (saline: 179 ± 38 ng/mL, *n* = 6; mepyramine: 89 ± 26 ng/mL, *n* = 6; *P* < .05 Tukey-Kramer), but not in *Apoe* ^−/−^ (saline: 206 ± 30 ng/mL, *n* = 8; mepyramine: 224 ± 10 ng/mL, *n* = 9) mice. There were an effect of genotype (*P* < .01) and a genotype x treatment interaction (*P* = .0474). Mepyramine also reduced plasma ACTH levels in wild-type mice (saline: 121 ± 20 pg/mL, *n* = 6; mepyramine: 77 ± 9 pg/mL, *n* = 6; *P* < .05 Tukey-Kramer), but not in *Apoe* ^−/−^ mice (saline: 57 ± 5 pg/mL, *n* = 8; mepyramine: 62 ± 11 pg/mL, *n* = 9). These data show that in *Apoe* ^−/−^ mice, mepyramine does not reduce measures of anxiety by inhibiting the HPA axis response. The dissociation of the effects of H_1_ receptor blockade on anxiety from those on the HPA axis in *Apoe* ^−/−^ and wild-type mice and the differential effects of H_3_ receptor blockade on novel object recognition and anxiety in *Apoe* ^−/−^, but not wild-type, mice suggest that differential pharmacokinetic profiles of histaminergic drugs in the two genotypes do not underlie the behavioral results.

There are no differences in H_1_ receptor expression in young (3–5-month old) *Apoe* ^−/−^ and wild-type mice. Saturation analysis with [^3^H] mepyramine in brain regions implicated in cognition or emotion [17] showed similar total number of receptors (B_max_ in nM) in the amygdala (wild-type: 103.4 ± 13.16; *Apoe* ^−/−^: 120.9 ± 20.92), cortex (wild-type: 126.5 ± 8.472; *Apoe* ^−/−^: 170.0 ± 13.02), hippocampus (wild-type: 104.0 ± 10.13; *Apoe* ^−/−^: 98.66 ± 11.03), and hypothalamus (wild-type: 218.7 ± 22.33; *Apoe* ^−/−^: 159.4 ± 29.00) of *Apoe* ^−/−^ and wild-type mice and similar binding affinities of [^3^H]-mepyramine in each brain region.

## 6. HUMAN APOE ISOFORMS AND MEASURES OF ANXIETY

We hypothesized that human apoE isoforms have differential effects on measures of anxiety in adult (6–8 months of age) *Apoe* ^−/−^ mice expressing human apoE3 or apoE4 at similar levels. *Apoe* ^−/−^ male mice without human apoE expression and apoE4 mice showed increased measures of anxiety in the elevated plus maze, whereas apoE3 male mice behaved like wild-type controls (Table 1). These differential effects of apoE isoforms on anxiety were age-dependent and not seen in young (2–4-month-old) male mice.

The isoform-specific effects of apoE are independent of the cellular source of apoE. When anxiety levels in the elevated plus maze were assessed in a cohort of 6-month-old GFAP-apoE male mice, in which the expression of apoE3 or apoE4 is targeted to astrocytes, GFAP-apoE3, but not GFAP-apoE4, male mice showed less measures of anxiety in the elevated plus maze than *Apoe* ^−/−^ mice (Table 2). Similar results were seen in the elevated zero maze, in which the mouse does not need to turn around in the open areas in order to return to the closed areas [24].

**Figure 3:**
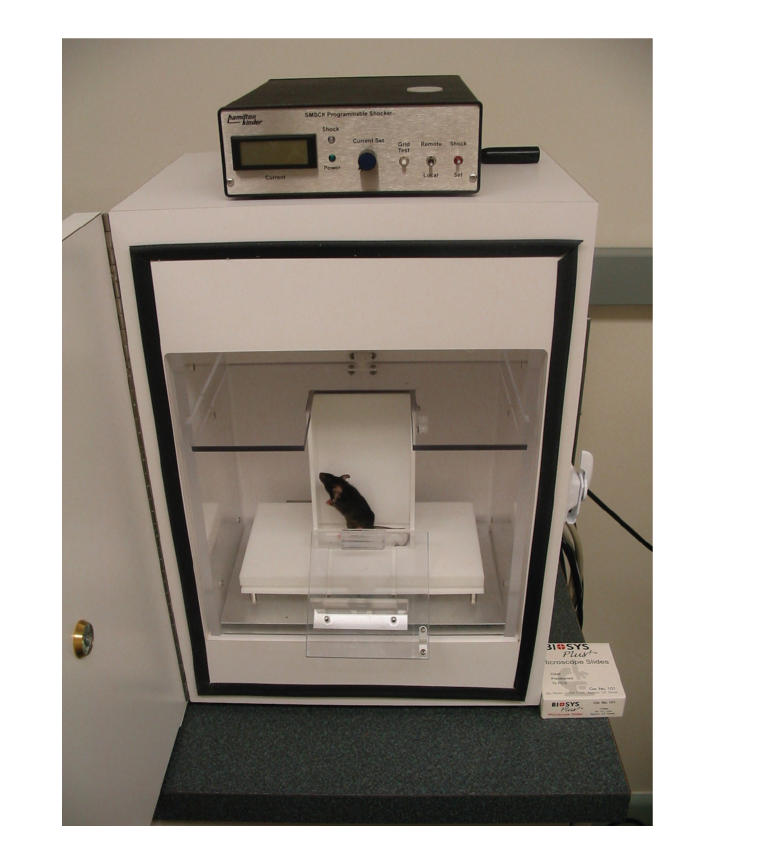
Acoustic startle: the mice are placed on a sensing platform and their response to acoustic stimuli is recorded.

The elevated plus maze is different from tests that involve unavoidable anxiety-provoking stimuli, such as acoustic stimuli. By assessing the acoustic startle response, we determined that apoE also has age- and isoform-specific effects on anxiety elicited by unavoidable acoustic stimuli. To measure startle reflex, we used the SM100 startle monitor system (Hamilton-Kinder) (Figure 3). At 3 months of age, there were no effects of apoE isoforms on the acoustic startle response. However, there were differential effects of apoE isoforms on the acoustic startle response at 6 months of age. *Apoe* ^−/−^ mice showed a higher acoustic startle response than age-matched wild-type mice. This was not present in apoE3 mice and was present to a lesser extent in apoE4 mice. There was a difference in acoustic startle response between apoE3 and apoE4 mice and between *Apoe* ^−/−^ and apoE3 or apoE4 mice [25]. Thus, the differential effects of apoE isoforms on measures of anxiety are not limited to avoidable anxiety-provoking stimuli. There were no differences in hearing threshold or fear-potentiated startle in the 6-month-old male groups.

## 7. DEXAMETHASONE SUPPRESSION AND APOE4

Impaired suppression of cortisol levels after administration of dexamethasone is reported in AD [26]. Therefore, we examined whether dexamethasone could suppress plasma corticosterone in adult apoE transgenic mice [25]. Mice were injected with dexamethasone (0.1 mg/kg) or saline between 9:00 a.m. and 10:00 a.m., and trunk blood was collected 6 hours later [27]. Compared to saline, dexamethasone suppressed plasma corticosterone levels in wild-type, *Apoe* ^−/−^, and apoE3 mice but dexamethasone suppression was impaired in apoE4 mice. The impaired dexamethasone suppression in the apoE4 mice might relate to other perturbations of cortisol responsivity observed in AD, including reduced cortisol-mediated regulation of hippocampal glucose metabolism [28] and dexamethasone sensitivity of peripheral blood nuclear cells [29].

## 8. THE AMYGDALA AND APOE4

The differential effects of apoE on measures of anxiety were associated with neuropathological alterations in the central nucleus of the amygdala, which plays an important role in the regulation of anxiety. Compared to wild-type mice, Apoe^−/−^ and apoE4, but not apoE3, mice had lower levels of microtubule-associated protein (MAP) 2-positive neuronal dendrites (*P* < .05). These changes were age-dependent. Three-month-old wild-type and Apoe^−/−^ mice had similar levels of MAP 2-positive neuronal dendrites. Interestingly, in nondemented human subjects [30] and in AD subjects [31], amygdala atrophy increased with increasing *ε*4 allele dose. However, other studies did not find effects of the *ε*4 allele on amygdala atrophy. This might relate to differences in the mean age of the subjects in the different studies and a decrease in the effect of *ε*4 with advanced age.

## 9. DIFFERENTIAL EFFECTS OF APOE ISOFORMS ON MEASURES OF ANXIETY IN PRAD PATIENTS

Consistent with the mouse studies, apoE also has isoform-dependent effects on measures of anxiety in probable AD (PRAD) patients [25]. Diagnosis of probable AD was made in each case according to NINDS-ADRDA criteria [32]. Cornell depression scale and neuropsychiatric inventory (NPI) were recorded for all subjects (mean age ± SEM; all subjects: 73 ± 1 years; *ε*3/*ε*3: 75 ± 3 years; *ε*3/*ε*4: 73 ± 2 years; *ε*4/*ε*4: 71 ± 2 years). Subjects were nonsmokers in good general health and free of past or present major psychiatric or neurological disorders (other than AD). Male *ε*4/*ε*4 subjects had higher anxiety scores than gender-matched *ε*3/*ε*3 subjects (*P* < .05). In males, but not in females, subjects with *ε*4/*ε*4 had also higher anxiety scores than those with *ε*3/*ε*4, suggesting that apoE3 can antagonize the effects of apoE4 on measures of anxiety in males but not in females. The anxiety scores did not correlate with the mini-mental state exam (MMSE) scores. Compared to *ε*3/*ε*3 male subjects, sleep disturbances were lower in *ε*4/*ε*4 (*P* < .01) and *ε*3/*ε*4 (*P* < .05) male subjects. Thus, sleep disturbances did not correlate or contribute to anxiety scores. ApoE did not have isoform-dependent effects on apathy or depression scores.

## 10. CONCLUSIONS

ApoE isoforms have differential effects on measures of anxiety in *Apoe* ^−/−^ mice expressing human apoE3 or apoE4 at similar levels and in PRAD subjects. The *ε*4 allele is also associated with depression in some [33–35] but not other [36–40] studies. As noncognitive behavioral changes are the major cause of institutionalization of AD patients and a major concern for their caregivers, more research aiming at increasing our understanding of mechanisms underlying these behavioral changes is needed to advance treatment strategies to reduce these changes.

## Figures and Tables

**Figure 2:**
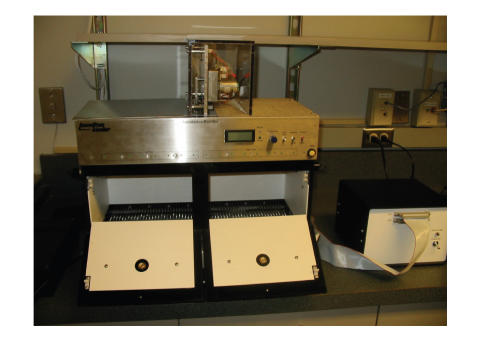
Passive avoidance: the mice are trained to avoid the preferred dark compartment by paring it with an aversive stimulus.

**Table 1:** Elevated plus-maze performance of 6–8-month-old NSE-apoE mice.

Genotype	Distance moved in closed arms (inches)	Time in closed arms (s)	Ratio time in open arms/time in open + time in closed arms^1^

Wild type (*n* = 8)	1003 ± 76	4962 ± 85	0.078 ± 0.015*
*Apoe* ^−/−^ (*n* = 37)	824 ± 30	5069 ± 113	0.035 ± 0.010
apoE3 (*n* = 27)	917 ± 40	4697 ± 100	0.087 ± 0.013**
apoE4 (*n* = 17)	886 ± 68	5204 ± 190	0.022 ± 0.007
apoE3/E4 (*n* = 9)	825 ± 66	4807 ± 77	0.053 ± 0.008

^1^There was a significance of genotype on ratio time in open arms/time in open + time in closed arms (*P* = .0094).

*P < .05, wild-type versus *Apoe* ^−/−^, apoE4, or apoE3/E4.

**P < .05 versus *Apoe* ^−/−^ and apoE3/E4, and *P* < .01 versus apoE4.

**Table 2:** Elevated plus-maze performance in 6-month-old GFAP-apoE male mice.

Genotype (line)	Distance moved in	Time in closed	Ratio time in open arms/time
	closed arms (inches)	arms (s)	in open + time in closed arms

*Apoe* ^−/−^ (*n* = 34)	1050 ± 43	475 ± 12	0.058 ± 0.012
apoE3 (127) (*n* = 11)	1137 ± 59	417 ± 14	0.174 ± 0.022*
apoE4 (129) (*n* = 4)	1194 ± 69	479 ± 34	0.044 ± 0.024
apoE4 (130) (*n* = 9)	1039 ± 36	475 ± 24	0.042 ± 0.016

*P < .05 versus *Apoe* ^−/−^ mice and *P* < .01 versus apoE4 (129) and apoE4 (130).
